# *Femorbiona* gen. nov., a new genus of sac spiders (Araneae, Clubionidae) from Southeast Asia

**DOI:** 10.3897/zookeys.1052.66803

**Published:** 2021-07-30

**Authors:** Jianshuang Zhang, Hao Yu, Shuqiang Li

**Affiliations:** 1 School of Life Sciences, Guizhou Normal University, Guiyang, Guizhou, China Guizhou Normal University Guiyang China; 2 School of Biological Sciences, Guizhou Education University, Guiyang, Guizhou, China Guizhou Education University Guiyang China; 3 Institute of Zoology, Chinese Academy of Sciences, Beijing, China Institute of Zoology, Chinese Academy of Sciences Beijing China

**Keywords:** China, morphology, new species, new combination, Vietnam, taxonomy

## Abstract

A new genus of Clubionidae Wagner, 1887, *Femorbiona* Yu & Li, **gen. nov.**, is described, with *Clubiona brachyptera* Zhu & Chen, 2012 (♂♀; Hainan, China) as the type species. Three species are included in *Femorbiona***gen. nov.**: *F. brachyptera***comb. nov.**, *F. phami* Yu & Li, **sp. nov.** (♂♀; Hai Phong, Vietnam), and *F. shenzhen* Yu & Li, **sp. nov.** (♂♀; Guangdong, China).

## Introduction

Clubionidae Wagner, 1887 is a relatively large family with 653 valid species distributed worldwide (Li 2020; WSC 2021). More than 80% of the species are currently assigned to the presumptively paraphyletic genus, *Clubiona* Latreille, 1804 (Marusik and Omelko 2018; Zhang and Yu 2020; Zhang et al. 2021). Deeleman-Reinhold (2001) provided a thorough analysis of the family from Southeast Asia, with eight genera and 57 species. In the same book, she also divided Southeast Asia *Clubiona* sensu lato into five species groups: *C. corticalis* group (corresponds to *Atalia* Thorell, 1887 and *Paraclubiona* Lohmander, 1944), *C. hystrix* group (corresponds to *Hirtia* Thorell, 1881), *C. japonica* group (corresponds to *Tolophus* Thorell, 1891 and *Japoniona* Mikhailov, 1990), *C. pahilistapyasea* group, and *C. pteronetoides* group (Marusik and Omelko 2018).

While examining spiders collected from southern China and Vietnam, we came across specimens that are reported here as belonging to two new clubionid species. Both possess several characters associated with a *Clubiona* species from Hainan, China, *C. brachyptera* Zhu & Chen, 2012, which was assigned to the *C. corticalis* group in the original paper (Zhu et al. 2012). The three species share a distinct set of characters and can be easily separated from all other genera of Clubionidae, in particular *Clubiona* sensu stricto (type *Araneus pallidulus* Clerck, 1757) and the type species of *Paraclubiona* and *Atalia* (two available generic names for the *corticalis* group, currently considered junior synonyms of *Clubiona*).

Thus, we established a new genus, *Femorbiona* Yu & Li, gen. nov., to accommodate the three species. The goal of this paper is to provide a description of the new genus and two new species, as well as a redescription of *C. brachyptera*, chosen as the type species of the new genus.

## Materials and methods

Specimens are deposited in the Institute of Zoology, Chinese Academy of Sciences (**IZCAS**) in Beijing (curator: Jun Chen), and the Museum of Hubei University (**MHU**) in Wuhan (curator: Jian Chen). Specimens were examined using both Leica M205C and Olympus SZX7 stereo-microscopes. The male and female copulatory organs were examined and illustrated after dissection. Left male palps are illustrated unless otherwise indicated (photos of the right palp were horizontally inverted to allow for ease of comparison). Epigynes were removed and cleared in lactic acid or a warm 10% potassium hydroxide (KOH) solution. Some vulvae were imaged after being embedded in Arabic gum. Images were captured with a Canon EOS 70D digital camera mounted on an Olympus CX41 compound microscope and assembled using Helicon Focus 6.80 image stacking software. All measurements were obtained using an Olympus SZX7 stereomicroscope and are given in millimetres. Eye diameters were taken from the widest distance. The total body length does not include chelicerae or spinnerets. Leg lengths are given as total length (femur, patella + tibia, metatarsus, tarsus). Terminology in the text and figure legends follows Yu et al. (2017), Zhang et al. (2018), Yu and Li (2019a, b), Zhang and Yu (2020), and Zhang et al. (2021).

References to figures in the cited papers are listed in lowercase (fig. or figs); figures from this paper are noted with an initial capital (Fig. or Figs). Abbreviations used in the text and figures are as follows:

**AER** anterior eye row

**ALE** anterior lateral eyes

**AME** anterior median eyes

**AME–AME** distance between AMEs

**AME–ALE** distance between AME and ALE

**BS** bursa

**C** conductor

**CO** copulatory opening

**E** embolus

**EB** embolic base

**FA** femoral apophysis

**H** hood

**MOQ** median ocular quadrangle

**MOQA**MOQ anterior width

**MOQL** length of MOQ

**MOQP**MOQ posterior width

**PER** posterior eye row

**PLE** posterior lateral eyes

**PME** posterior median eyes

**PME–PME** distance between PMEs

**PME–PLE** distance between PME and PLE

**PPA** prolateral patellar apophysis

**SP** spermatheca

**VTA** ventral tibial apophysis

## Taxonomy

### Family Clubionidae Wagner, 1887

#### Femorbiona

Taxon classificationAnimaliaAraneaeClubionidae

Yu & Li gen. nov.

C5B4D2E5-E51B-51BC-9BC2-63442FB27BE3

http://zoobank.org/37400162-2D7D-4168-8C80-34422CC75BF2

##### Type species.

*Clubiona brachyptera* Zhu & Chen, 2012 from Hainan, China.

##### Diagnosis.

*Femorbiona* gen. nov. differs from *Clubiona* sensu stricto by: a femoral apophysis (FA) and prolateral patellar apophysis (PPA) on the male palp (vs. lacking in *Clubiona*); male palpal tibia with a ventral apophysis (VTA) but without a retrolateral apophysis (RTA) (vs. VTA absent and RTA well developed in *Clubiona*); an inflated tegulum (vs. tegulum relatively flat in *Clubiona*); an indistinct, non-meandering sperm duct (vs. distinct and meandering in *Clubiona*). Females of *Femorbiona* gen. nov. differ from those of *Clubiona* sensu stricto by having tubular spermathecae (vs. subglobular or oval) and shorter copulatory ducts.

The male of *Femorbiona* gen. nov. also resembles species of the *C. corticalis* group by the enlarged and protruded tegulum and indistinct sperm duct, but it can be easily distinguished by the femoral and patellar apophyses (Fig. 7) (vs. palpal femur and patella unmodified in *corticalis*-group species) and the absence of an RTA (vs. present in *corticalis*-group species). The female of *Femorbiona* gen. nov. can be easily separated from all *corticalis*-group species by the posteriorly or centrally located copulatory openings (vs. anteriorly located).

##### Description.

Small to medium sized with body length 2.70–3.79 in males and 2.80–3.99 in females; carapace 1.60–1.70 long in males and 1.39–1.70 in females. Prosoma in profile highest just behind fovea, gradually sloping to pars thoracica, ca. 1.5–1.8 × longer than high; carapace smooth, with short, fine setae, uniformly yellowish brown or yellowish orange, slightly darker anteriorly; fovea short, longitudinal, ca. 2 × diameter of PME. Sternum yellowish, anterior edge truncate, lateral margin with brown extensions fitting intercoxal concavities, posterior region strongly protruded between coxae IV. Female palp without claw, distally with erect, thin, dark bristles. Chelicera uniformly coloured as ocular region, consisting of a coniform base and claw-shaped fang, promargin with more than five teeth, retromargin with more than four teeth. Labium ca. 1.5–1.6 × longer than wide, nearly trapezoidal, concave laterally. Maxillae depressed posteriorly, slightly convergent anteriorly, with dense scopulae on inner margin. Legs uniformly coloured as sternum, with darker femora and coxae I; all femora with four or five dorsal spines; all patellae with none or one dorsal or retrolateral spine; tibiae I–II with two pairs of ventral spines; metatarsi I–II with a pair of spines; tibiae and metatarsi of posterior legs with numerous spines, spination variable on tibiae III–IV and metatarsi III–IV. Leg formula 4213. Abdomen elongate-oval, 1.8–2 × longer than wide, > 2 × longer than high, uniformly coloured. Spinnerets: six, arranged as in all other clubionid spiders. Male palp: femur 2.8–3.2 × longer than wide, retrolaterally with apophysis, usually located proximally, longer or equal to femur diameter; patella modified, with prolateral apophysis, distinctly longer than tibia; tibia cup shaped, slightly wider than long in ventral view, with small, weak ventral apophysis; cymbium oval, ca. 2 × longer than wide; bulb oval, ca 1.6–1.8 × longer than wide; sperm duct indistinct, S-shaped in ventral view, broad part terminating at ca. 1 o’clock position; tegular apophysis absent; conductor small and weakly sclerotised in type species, absent in other species; embolus located prolatero-distally, embedded in tegulum, embolic base an enlarged tubercle, gradually tapered toward apex, embolic tip needle-like or claw shaped. Epigyne with distinct hood, or pocket-like lateral chitinous structures; copulatory openings located posteriorly, separated by 1–4 diameters; spermathecae tubular; bursae oblong, hyaline, much bigger than spermathecae.

##### Composition.

*Femorbiona brachyptera* (Zhu & Chen, 2012) comb. nov., *F. phami* sp. nov. and *F. shenzhen* sp. nov.

##### Etymology.

The generic name is derived from *Clubiona* and the unique presence of a femoral apophysis on the male palp; feminine in gender.

##### Comments.

There are approximately ten more clubionid species assigned to the *Clubiona corticalis* group that have palpal and femoral apophyses in males. A review of these species is beyond the scope of this work; however, the following may belong to the new genus: *C. femorocalcarata* Huang & Chen, 2012, *C. globosa* Wang, Chen & Zhang, 2018, *C. kayashimai* Ono, 1994, *C. pollicaris* Wu, Zheng & Zhang, 2015, *C. qiyunensis* Xu, Yang & Song, 2003.

#### Femorbiona brachyptera

Taxon classificationAnimaliaAraneaeClubionidae

(Zhu & Chen, 2012) comb. nov.

CE196E9B-CE68-5B06-A6F9-E59010482909

Figs 1 , 2 , 7A

Clubiona brachyptera Zhu & Chen, in Zhu et al. 2012: 53, figs 1–12 (♂♀).

##### Type material.

***Holotype*** ♂ (**MHU**), ***paratypes*** 3♂4♀ (**MHU)**, China: Hainan Island: Qiongzhong County, Mt. Limu, natural forest, 19°16.012'N, 109°46.048'E, ca. 650 m, 28.VIII.2010, J. Liu and H.Q. Ren leg.

##### Diagnosis.

*Femorbiona brachyptera* resembles the species *F. phami* sp. nov. and *F. shenzhen* sp. nov. by the general shape of the palp and the endogyne. Males of *F. brachyptera* can be easily distinguished from the two congeners by the weakly sclerotised conductor (vs. absent) (cf. Fig. 7A and Fig. 7B, C) and the abdomen dorsally marked with numerous brown spots (vs. abdomen without distinct pattern or markings) (cf. Fig. 2E and Figs 4E, 6E). Females of *F. brachyptera* can be easily recognised by the strongly convoluted dorsal part of the spermathecae, which follows a double S-shaped course (Fig. 2C, D) (vs. moderately convoluted, following an S-shaped course in *F. phami* sp. nov., as in Fig. 4C, D; not convoluted, following a C-shaped course in *F. shenzhen* sp. nov., as in Fig. 6C, D).

##### Description.

**Male.** Paratype (Fig. 2E, F): Total length 3.14; carapace 1.43 long, 1.04 wide; opisthosoma 1.71 long, 0.92 wide. Carapace light orange, uniformly coloured, without distinct pattern. Eyes: AER slightly recurved, PER slightly wider than AER, almost straight in dorsal view. AME dark, other eyes light; with black rings. Eye sizes and interdistances: AME 0.07, ALE 0.11, PME 0.08, PLE 0.10, AME–AME 0.06, AME–ALE 0.03, PME–PME 0.17, PME–PLE 0.08, MOQL 0.24, MOQA 0.21, MOQP 0.38. Chelicerae coloured as carapace, with 5 promarginal and 4 retromarginal teeth. Labium and endites coloured as chelicerae, longer than wide. Sternum yellowish white. Legs coloured as sternum, without distinct markings. Leg measurements: I 2.90 (0.81, 1.19, 0.52, 0.38), II 3.24 (0.97, 1.30, 0.60, 0.37), III 2.62 (0.80, 0.67, 0.25, 2.62), IV 3.90 (1.14, 1.34, 1.04, 0.38). Abdomen elongate-oval, dorsally and laterally marked with numerous brown spots, dorsum centrally with one pair of inconspicuous muscle depressions; venter white with no distinct pattern.

**Figure 1.**
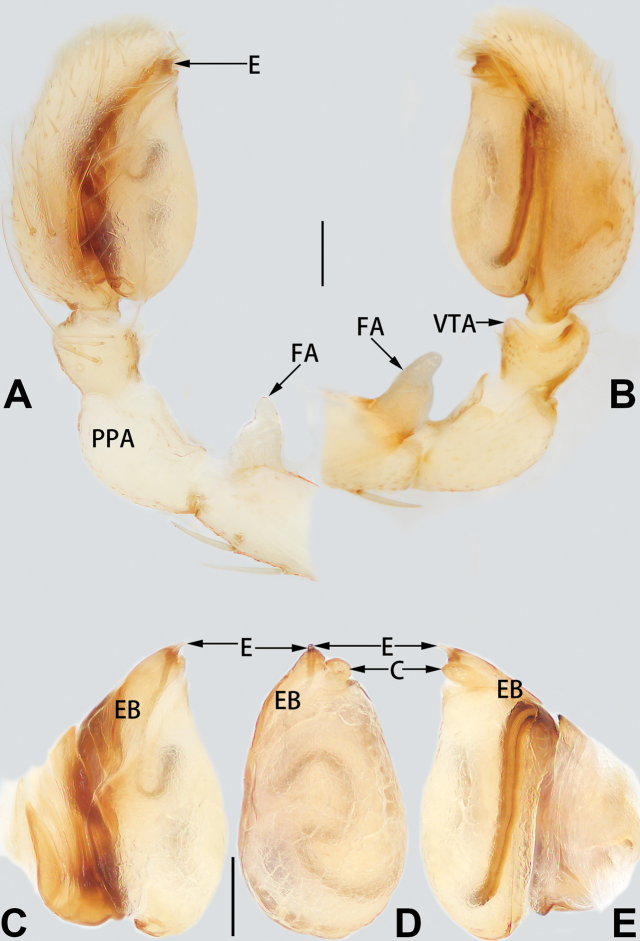
*Femorbiona brachyptera*, paratype male palp **A** prolateral view **B** retrolateral view **C** bulb, prolateral view **D** bulb, ventral view **E** bulb, retrolateral view. Abbreviations: C = conductor; E = embolus; EB = embolic base; FA = femoral apophysis; PPA = prolateral patellar apophysis; VTA = ventral tibial apophysis. Scale bars: 0.1 mm.

Palp (Figs 1A–E, 7A): Femur with short retrolateral apophysis originating proximally; FA ca. 1/3 length of femur, thin distally, wide basally, shaped like a short wing of a bird or dorsal fin of a fish in retrolateral view. Patella 1.75 × longer and 1.3–1.5 × wider than tibia, prolaterally with round apophysis. Tibia cup shaped, relatively short, < 1/4 of cymbium length, ventro-retrolateral tibial apophysis subtriangular, with blunt tip. Tegulum elongate, oval, prolapsed, ca. 1.7 × longer than wide; sperm duct indistinct, S-shaped in ventral view. Embolus claw shaped, base an enlarged tubercle, originating on prolateral flank (approximately 10 o’clock on tegulum), gradually tapered toward tip; embolar apex needle-like, < 1/6 of base length, apex sharp, ventro-distally pointed. Conductor sclerotised, short, 1/14–1/13 of tegulum length, elliptical, slightly curved around embolus.

**Figure 2.**
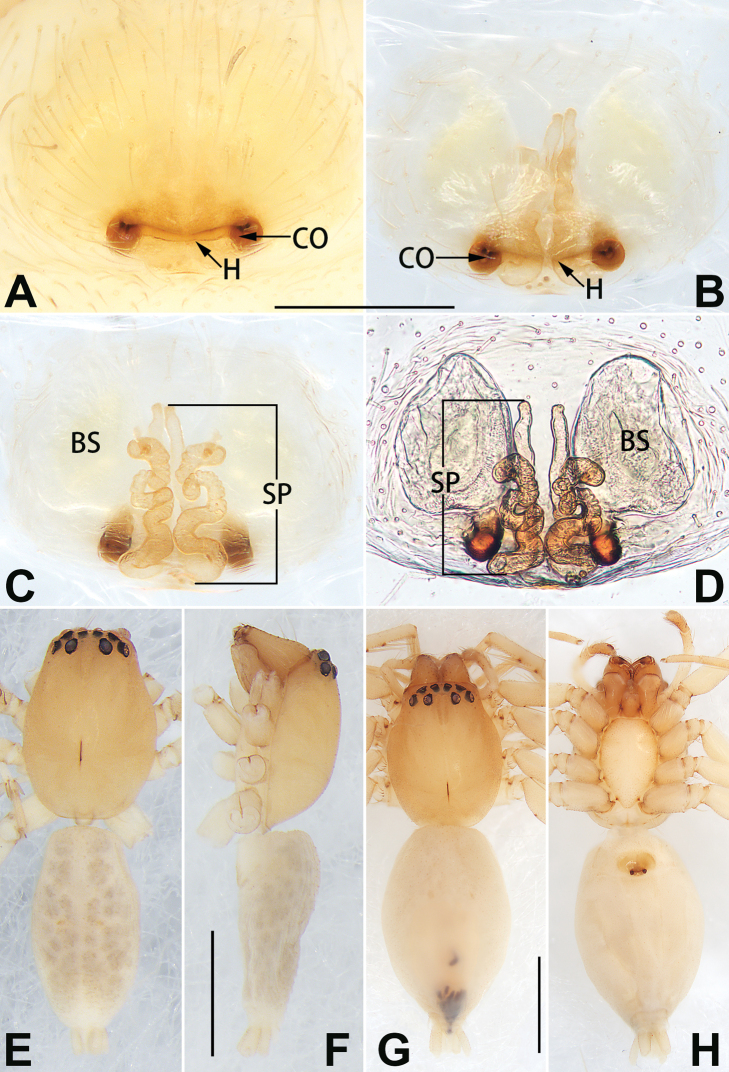
*Femorbiona brachyptera*, female and male paratypes, epigyne (**A–D**), male habitus (**E, F**) and female habitus (**G, H**) **A** intact, ventral view **B** cleared, ventral view **C** cleared, dorsal view **D** cleared, dorsal view **E** dorsal view **F** lateral view **G** dorsal view **H** ventral view. Abbreviations: BS = bursa; CO = copulatory opening; H= hood; SP = spermatheca. Scale bars: 0.1 mm (**A–D**); 1 mm (**E–H**).

**Female.** Paratype (Fig. 2G, H): total length 3.74; carapace 1.46 long, 1.02 wide; opisthosoma 2.28 long, 1.23 wide. Eye sizes and interdistances: AME 0.06, ALE 0.09, PME 0.09, PLE 0.08, AME–AME 0.06, AME–ALE 0.06, PME–PME 0.17, PME–PLE 0.07, MOQL 0.19, MOQA 0.19, MOQP 0.34. Legs yellowish white, without distinct markings. Leg measurements: I 2.68 (0.76, 1.07, 0.48, 0.37), II 2.85 (0.82, 1.17, 0.53, 0.32), III 2.53 (0.75, 0.87, 0.61, 0.29), IV 3.75 (1.11, 1.27, 1.00, 0.436). Similar to males but slightly larger and darker.

Epigyne (Fig. 2A–D). Epigynal plate ca. 1.35 × wider than long, margins slightly rebordered; arrangement of vulva imperceptible through transparent integument in ventral view. Hood located posteriorly on epigynal plate, ca. 1/2 of epigyne width, slightly procurved, V-shaped or horizontal. Copulatory openings circular, located at lateral border of hood, separated by ca. 3 diameters. Copulatory ducts thick, covered by large spermathecae in dorsal view, directed anteriorly, then connected to spermathecae. Spermathecae long, tubular, sinuous, dorsal part strongly convoluted, following a double S-shaped course, ventral part almost parallel, ascending > 2/3 the length of epigynal plate. Bursae oval, translucent, surface smooth, separated by ca. 0.5 × diameters, ca. 1.5 × longer than wide.

##### Distribution.

China (Hainan).

#### Femorbiona phami

Taxon classificationAnimaliaAraneaeClubionidae

Yu & Li sp. nov.

FABBED9E-C037-5C75-BB6A-0DDC4F7273DD

http://zoobank.org/B685CC15-07F5-431F-8A9B-B9A69906A55D

Figs 3 , 4 , 7B

##### Type material.

***Holotype*** ♂ (IZCAS Ar 34724), VIETNAM: Hai Phong: Cat Ba National Park: disturbed forest, 20°48.258'N, 107°00.581'E, ca. 80 m, 15.VII.2008, D.S. Pham leg. ***Paratypes***: 1♀ (IZCAS Ar 34725), same locality and collector as holotype, 20°48.249'N, 107°00.016'E, ca. 80 m, 16.VII.2008.

##### Diagnosis.

**Figure 3.**
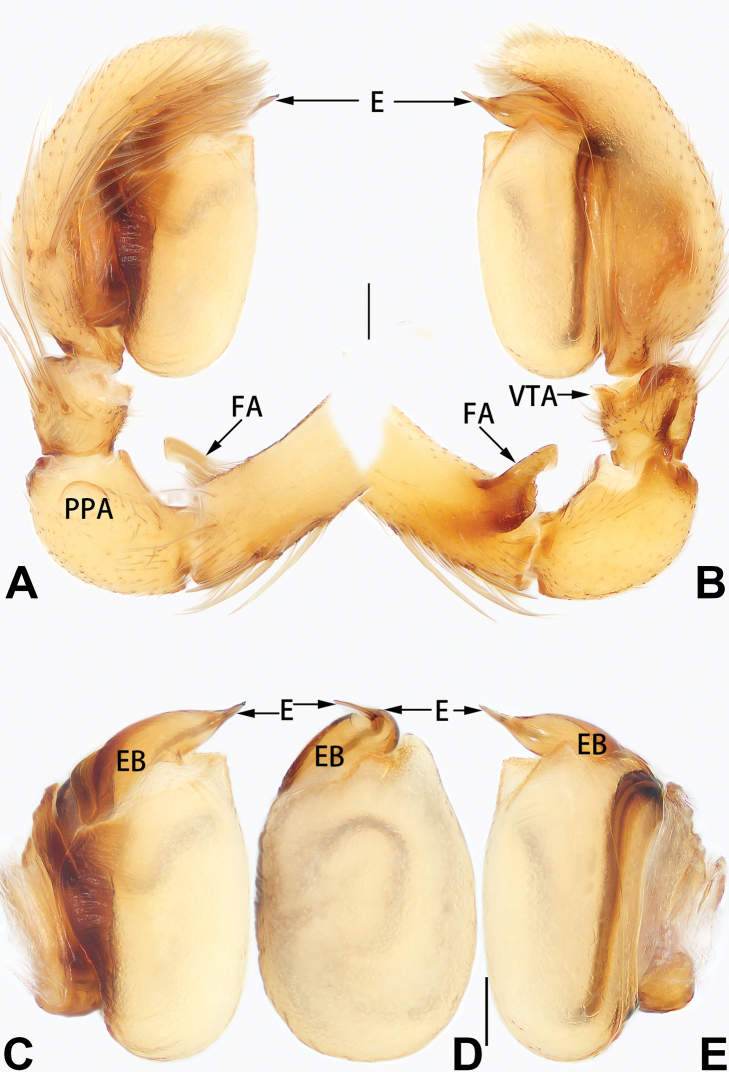
*Femorbiona phami* sp. nov., holotype male palp **A** prolateral view **B** retrolateral view **C** bulb, prolateral view **D** bulb, ventral view **E** bulb, retrolateral view. Abbreviations: E = embolus; EB = embolic base; FA = femoral apophysis; PPA = prolateral patellar apophysis; VTA = ventral tibial apophysis. Scale bars: 0.1 mm.

**Figure 4.**
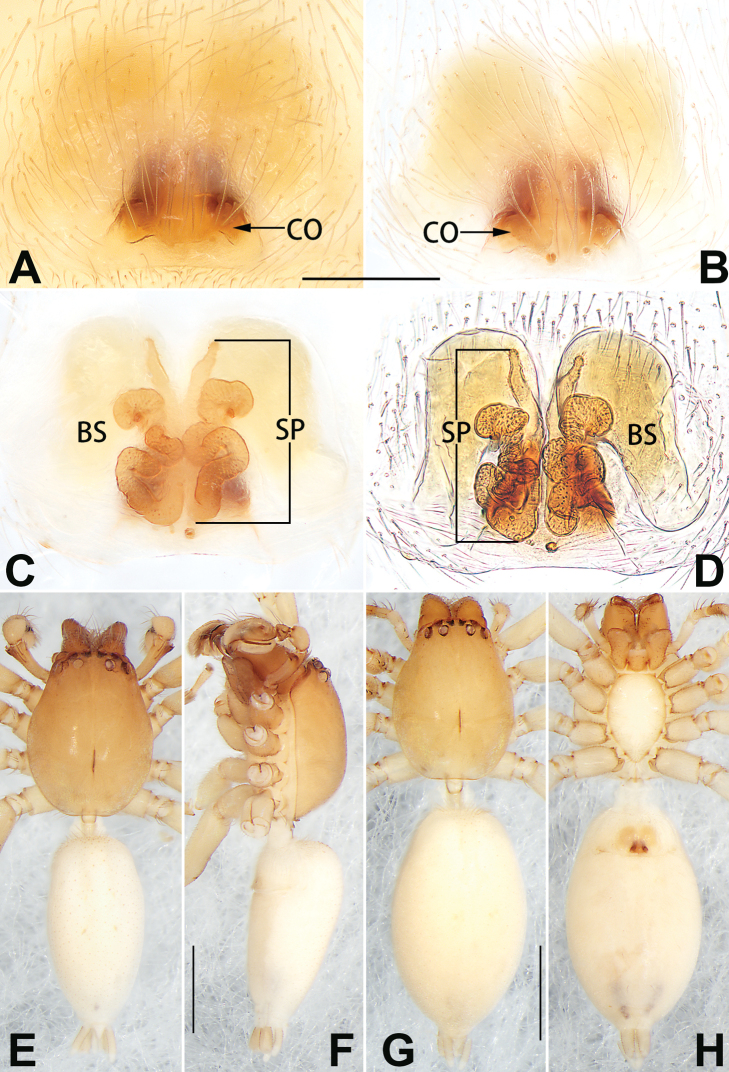
*Femorbiona phami* sp. nov., female paratype and male holotype, epigyne (**A–D**), male habitus (**E, F**) and female habitus (**G, H**) **A** intact, ventral view **B** cleared, ventral view **C** cleared, dorsal view **D** cleared, dorsal view **E** dorsal view **F** lateral view **G** dorsal view **H** ventral view. Abbreviations: BS = bursa; CO = copulatory opening; SP = spermatheca. Scale bars: 0.1 mm (**A–D**); 1 mm (**E–H**).

The males of *F. phami* sp. nov. are most similar to those of *F. brachyptera* (Zhu et al. 2012: 53, figs 2–4, 8–10; Figs 1A–E, 7A) by the general shape of the palpal femoral apophysis, which is shaped like a short bird wing in retrolateral view (cf. Fig. 1B and Fig. 3B), but can be distinguished from *F. brachyptera* by having: (1) the distal tip of the femoral apophysis slightly curved (vs. not curved) (cf. Fig. 7B and Fig. 7A); (2) in ventral view, the embolar apex relatively long, over 1/3 the length of the embolic base and pointed prolatero-distally (Figs 3D, 7B) (vs. relatively short tip, < 1/6 the length of the embolic base, pointed ventro-distally, as in Figs 1D, 7A); (3) conductor absent (vs. present) (cf. Figs 3C–E, 7B and Figs 1C–E, 7A). The female of *F. phami* sp. nov. can be easily distinguished from all other congeners by the slightly sclerotised and pocket-like copulatory openings and by the absence of an epigynal hood (Fig. 4A, B) (vs. 2 circular copulatory openings located at the lateral border of hood, as in Figs 2A, B, 6A, B).

##### Description.

**Male.** Holotype (Fig. 4E, F): Total length 3.51; carapace 1.66 long, 1.28 wide; opisthosoma 1.85 long, 1.01 wide. Carapace orange, pars cephalica darker in ocular area, without distinct pattern. Eyes: both AER and PER slightly recurved in dorsal view. AME dark, other eyes light; with black rings. Eye sizes and interdistances: AME 0.07, ALE 0.13, PME 0.10, PLE 0.12, AME–AME 0.08, AME–ALE 0.06, PME–PME 0.20, PME–PLE 0.12, MOQL 0.22, MOQA 0.21, MOQP 0.38. Chelicerae slightly darker than ocular area, with 6 promarginal and 5 retromarginal teeth. Labium and endites coloured as chelicerae, longer than wide. Sternum yellowish white. Legs coloured as sternum, without distinct markings. Leg measurements: I 3.56 (0.99, 1.48, 0.70, 0.39), II 4.11 (1.18, 1.69, 0.80, 0.44), III 3.27 (0.95, 1.17, 0.82, 0.33), IV 4.74 (1.32, 1.62, 1.36, 0.44). Abdomen elongate-oval, dorsally yellowish white, dorsum with 2 pairs of inconspicuous muscle depressions; venter white with no distinct pattern.

Palp (Figs 3A–E, 7B): Femur with short retrolateral apophysis originating proximally; FA ca. 1/3–1/4 length of femur, thin distally, wide basally. Patella 2 × longer and 1.3–1.5 × wider than tibia, with round, short prolateral apophysis. Tibia cup shaped, relatively short, < 1/3 of cymbium length, with papilliform, partly membranous, ventro-retrolateral apophysis. Tegulum elongate, oval, and inflated, ca. 1.6 × longer than wide; sperm duct indistinct in ventral view. Embolus somewhat helical, more or less Ƨ-shaped in ventral view, base an enlarged tubercle, inserted at approximately the 10–11 o’clock position on tegulum, gradually tapered toward tip; embolar apex needle-like, prolaterally pointed, over 1/3 of base length.

**Female.** Paratype (Fig. 4G, H): total length 3.99; carapace 1.84 long, 1.43 wide; opisthosoma 2.15 long, 1.07 wide. Eye sizes and interdistances: AME 0.09, ALE 0.13, PME 0.12, PLE 0.10, AME–AME 0.06, AME–ALE 0.06, PME–PME 0.12, PME–PLE 0.15, MOQL 0.27, MOQA 0.27, MOQP 0.48. Legs yellowish white, without distinct markings. Leg measurements: I 4.10 (1.18, 1.70, 0.80, 0.42), II 4.60 (1.33, 1.92, 0.93, 0.42), III 3.72 (1.11, 1.28, 0.93, 0.40), IV 5.31 (1.52, 1.86, 1.47, 0.46). Similar to males but slightly smaller and lighter.

Epigyne (Fig. 4A–D). Epigynal plate wider than long, anterior and lateral margins not rebordered; spermathecae and bursae indistinct, copulatory ducts easily visible through integument in ventral view. Copulatory openings large, pocket-like, located on chitinous structures at the postero-lateral portion of epigynal plate, separated by ca. 1 diameter. Copulatory ducts thick, covered by large spermathecae in dorsal view, directed anteriorly, then connected to spermathecae. Spermathecae long, tubular, sinuous. Bursae oblong, translucent, surface smooth, close together, ca. 2 × longer than wide.

##### Distribution.

Known only from the type locality, Hai Phong, Vietnam.

##### Etymology.

The specific name is a patronym after Dinh Sac Pham (Hanoi, Vietnam), collector of the type series; noun (name) in apposition.

#### Femorbiona shenzhen

Taxon classificationAnimaliaAraneaeClubionidae

Yu & Li sp. nov.

395D05E9-C083-5AAB-B69E-A22E4A1B5116

http://zoobank.org/F828E5CC-CEBA-4FBC-942F-3FF97438099B

Figs 5 , 6 , 7C , 8

##### Type material.

***Holotype*** ♂ (IZCAS-Ar 34726), CHINA: Guangdong Province: Shenzhen: Meilin Reservoir, 22°34.365'N, 114°0.400'E, ca. 100 m, 01.XI.2020, Q. Lu. leg. ***Paratype***: 1♀ (IZCAS-Ar 34727), same data as holotype.

##### Diagnosis.

**Figure 5.**
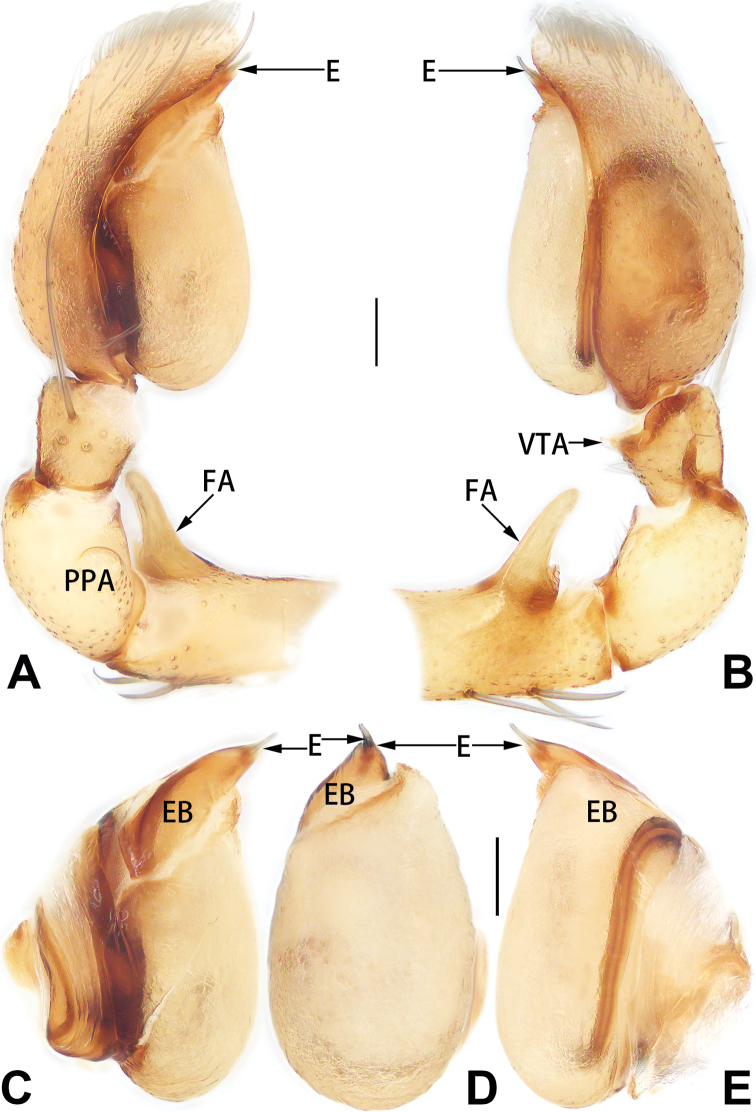
*Femorbiona shenzhen* sp. nov., holotype male palp **A** prolateral view **B** retrolateral view **C** bulb, prolateral view **D** bulb, ventral view **E** bulb, retrolateral view. Abbreviations: E = embolus; EB = embolic base; FA = femoral apophysis; PPA = prolateral patellar apophysis; VTA = ventral tibial apophysis. Scale bars: 0.1 mm.

**Figure 6.**
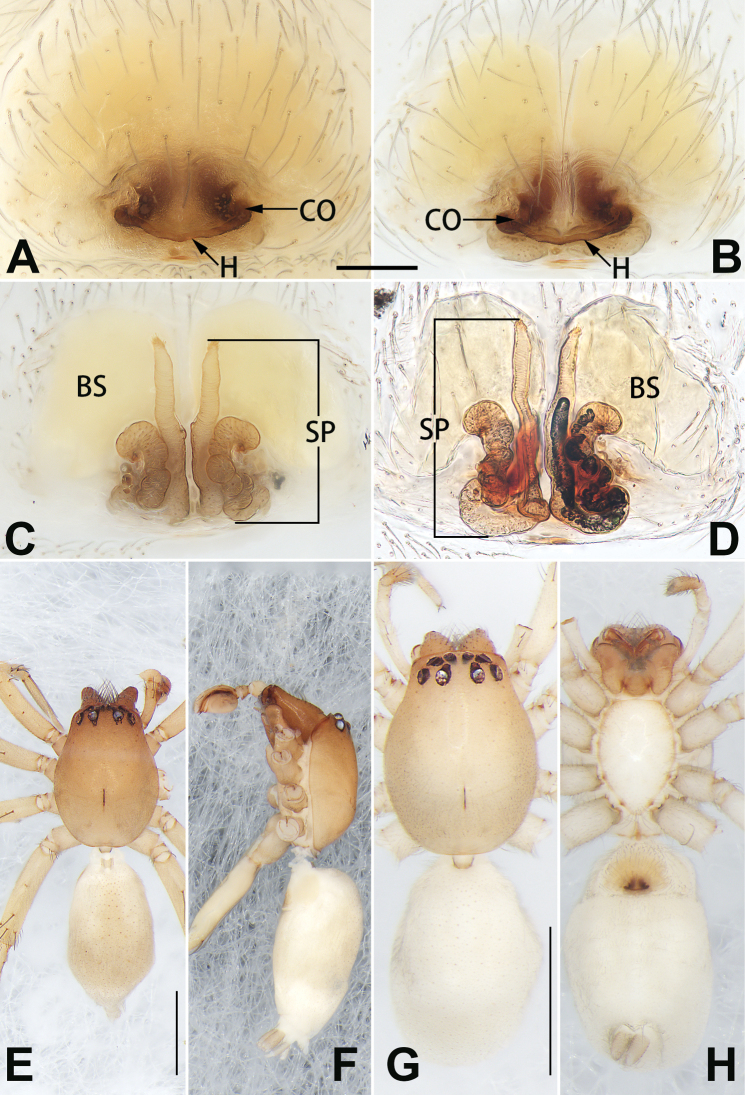
*Femorbiona shenzhen* sp. nov., male holotype and female paratype, epigyne (**A–D**), male habitus (**E, F**) and female habitus (**G, H**) **A** intact, ventral view **B** cleared, ventral view **C** cleared, dorsal view **D** cleared, dorsal view **E** dorsal view **F** lateral view **G** dorsal view **H** ventral view. Abbreviations: BS = bursa; CO = copulatory opening; H= hood; SP = spermatheca. Scale bars: 0.1 mm (**A–D**); 1 mm (**E–H**).

**Figure 7.**
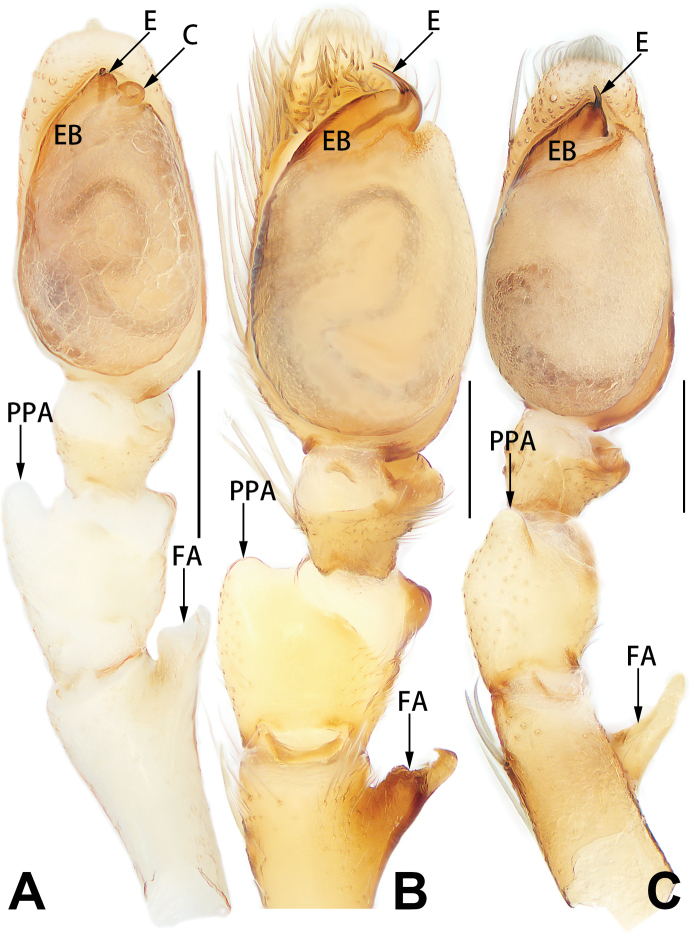
*Femorbiona* spp., left male palp, ventral view **A***F. brachyptera*, paratype **B***F. phami* sp. nov., holotype **C***F. shenzhen* sp. nov., holotype. Abbreviations: C = conductor; E = embolus; FA = femoral apophysis; EB = embolic base; PPA = prolateral patellar apophysis. Scale bars: 0.2 mm.

The male of *F. shenzhen* sp. nov. resembles that of *F. phami* sp. nov. (Figs 3A–E, 7B) by the embolus which consists of an enlarged base and needle-like apex (cf. Figs 5C–E, 7C and Figs 3C–E, 7B), but it differs by having: (1) the femoral apophysis finger shaped (Figs 5A, B, 7C) (vs. shaped like a short wing of a bird or dorsal fin of a fish, as in Fig. 3A, B); (2) in ventral view, the embolar apex pointed ventro-distally (Figs 5D, 7C) (vs. pointed prolatero-distally, as in Figs 3D, 7B). The female is similar to *F. brachyptera* (Zhu et al. 2012: 53, figs. 6, 7, 11, 12; Fig. 2A–D) by the V-shaped epigynal hoods (cf. Fig. 2A, B and Fig. 6A, B), but it differs by having: (1) the copulatory ducts easily visible through the epigynal plate in ventral view (Fig. 6A, B) (vs. indistinct, as in Fig. 2A, B); (2) the dorsal part of the spermathecae not strongly convoluted, following a C-shaped course (Fig. 6C, D) (vs. strongly convoluted, following a double S-shaped course, as in Fig. 2C, D).

##### Description.

**Figure 8.**
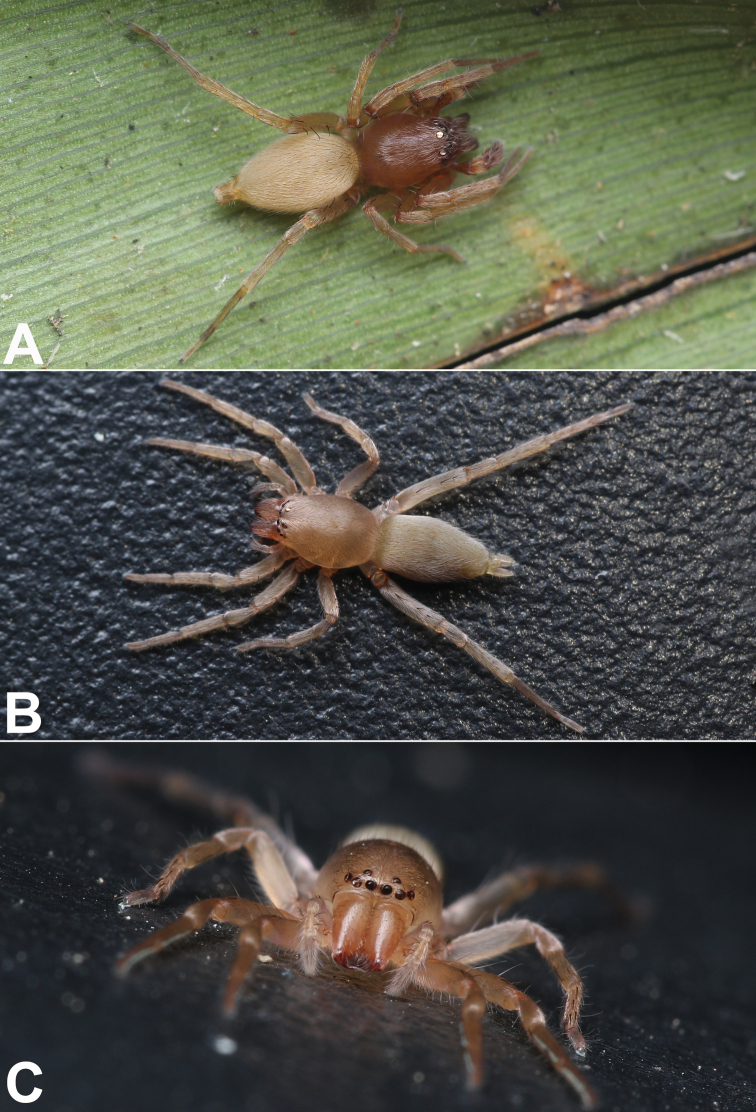
*Femorbiona shenzhen* sp. nov., male holotype (**A**) and female paratype (**B, C**), live specimens. Photographs by Qianle Lu (Shenzhen, Guangdong).

**Male.** Holotype (Figs 6E, F, 8A). Total length 3.79; carapace 1.70 long, 1.22 wide; opisthosoma 2.09 long, 1.02 wide. Colour in life dark brown with red-grey abdomen (Fig. 8A). Carapace light brown in ethanol (Fig. 6E, F), darker anteriorly, without distinct pattern, fovea black; cephalic region distinctly narrowed, cervical groove and radial grooves indistinct; tegument smooth, clothed with short, fine setae. Eyes: both AER and PER slightly recurved in dorsal view, latter wider than former. Eye sizes and interdistances: AME 0.09, ALE 0.12, PME 0.12, PLE 0.12, AME–AME 0.07, AME–ALE 0.04, PME–PME 0.20, PME–PLE 0.09, MOQL 0.27, MOQA 0.24, MOQP 0.43. Chelicerae robust, dark brown, with six promarginal and four retromarginal teeth. Sternum yellowish white, 0.94 long, 0.62 wide. Labium and endites coloured as carapace. Legs coloured as sternum, without distinct markings. Leg measurements: I 3.59 (1.03, 1.51, 0.68, 0.36), II 4.19 (1.21, 1.75, 0.82, 0.41), III 3.26 (0.97, 1.17, 0.81, 0.31), IV 4.75 (1.43, 1.60, 1.29, 0.43). Abdomen elongate-oval, without pattern; dorsum greyish, anteriorly slightly darker; venter uniformly grey.

Palp (Figs 5A–E, 7C). Femur with finger-shaped retrolateral apophysis originating proximally, apophysis < 1/3 length of femur. Patella 2 × longer and 1.3 × wider than tibia, with round prolateral apophysis. Tibia with small, partly membranous ventral apophysis. Bulb inflated, sperm duct indistinct in ventral view. Embolus needle-like, relatively short, < 1/10 of tegulum length, originating distally on tegulum, gradually tapered toward tip, apex sharp, ventrally pointed; embolic base an enlarged tubercle.

**Female.** Paratype (Figs 6G, H, 8B, C). Total length 2.86; carapace 1.39 long, 1.00 wide; opisthosoma 1.48 long, 0.97 wide. Eye sizes and interdistances: AME 0.06, ALE 0.09, PME 0.08, PLE 0.07, AME–AME 0.06, AME–ALE 0.06, PME–PME 0.15, PME–PLE 0.05, MOQL 0.23, MOQA 0.20, MOQP 0.32. Sternum 0.77 long, 0.50 wide. Leg measurements: I 2.52 (0.69, 1.04, 0.51, 0.28), II missing, III 2.48 (0.69, 1.00, 0.45, 0.34), IV 3.61 (1.09, 1.22, 1.01, 0.28). Similar to male but distinctly larger and lighter coloured.

Epigyne (Fig. 6A–D). Epigynal plate distinctly wider than long, through which bursae and copulatory ducts are conspicuous. Hood located posteriorly on epigynal plate, ca. 1/3 of epigyne width, posterior margin heavily sclerotised, slightly procurved, V-shaped. Copulatory openings at lateral border of hood, separated by ca. 3 diameters. Copulatory ducts thick, short, covered by large spermathecae in dorsal view. Spermathecae long, tubular, sinuous, with uniform thickness throughout. Bursae reniform, large, close together, ca. 1.5 × longer than wide, surface translucent, smooth.

##### Distribution.

Known only from the type locality, Shenzhen, Guangdong, China.

##### Etymology.

The species name is derived from the type locality; noun in apposition.

## Supplementary Material

XML Treatment for Femorbiona

XML Treatment for Femorbiona brachyptera

XML Treatment for Femorbiona phami

XML Treatment for Femorbiona shenzhen
